# Multi-Institutional Prospective Randomized Control Trial of Novel Intracorporeal Lithotripters: ShockPulse-SE *vs* Trilogy Trial

**DOI:** 10.1089/end.2020.1097

**Published:** 2021-09-14

**Authors:** Tim Large, Charles Nottingham, Ethan Brinkman, Deepak Agarwal, Andrea Ferrero, Michael Sourial, Karen Stern, Marcelino Rivera, Bodo Knudsen, Mitchel Humphreys, Amy Krambeck

**Affiliations:** ^1^ Department of Urology, Indiana University, Indianapolis, Indiana, USA.; ^2^ Department of Radiology, Mayo Clinic—Rochester, Rochester, Minnesota, USA.; ^3^ Department of Urology, Ohio State University, Columbus, Ohio, USA.; ^4^ Department of Urology, Mayo Clinic—Scottsdale, Scottsdale, Arizona, USA.; ^5^ Department of Urology, Northwestern University, Feinberg School of Medicine, Chicago, Illinois, USA.

**Keywords:** nephrolithiasis, percutaneous nephrolithotomy, lithotripter

## Abstract

***Introduction:*** Currently, there are multiple intracorporeal lithotripters available for use in percutaneous nephrolithotomy (PCNL). This study aimed to evaluate the efficiency of two novel lithotripters: Trilogy and ShockPulse-SE.

***Materials and Methods:*** This is a prospective multi-institutional randomized trial comparing outcomes of PCNL using two novel lithotripters between February 2019 and June 2020. The study assessed objective measures of stone clearance time, stone clearance rate, device malfunction, stone-free rates, and complications. Device assessment was provided through immediate postoperative survey by primary surgeons.

***Results:*** There were 100 standard PCNLs completed using either a Trilogy or ShockPulse-SE lithotrite. Using quantitative Stone Analysis Software to estimate stone volume, the mean stone volume was calculated at 4.18 ± 4.79 and 3.86 ± 3.43 cm^3^ for the Trilogy and ShockPulse-SE groups, respectively. Stone clearance rates were found to be 1.22 ± 1.67 and 0.77 ± 0.68 cm^3^/min for Trilogy *vs* ShockPulse-SE (*p* = 0.0542). When comparing Trilogy to ShockPulse-SE in a multivariate analysis, total operative room time (104.4 ± 48.2 minutes *vs* 121.1 ± 59.2 minutes *p* = 0.126), rates of secondary procedures (17.65% *vs* 40.81%, *p* = 0.005), and device malfunctions (1.96% *vs* 34.69%, *p* < 0.001) were less, respectively. There was no difference in final stone-free rates between devices.

***Conclusion:*** Both the Trilogy and ShockPulse-SE lithotripters are highly efficient at removing large renal stones. In this study, we noted differences between the two devices including fewer device malfunctions when Trilogy device was utilized. Clinical Trial ID number: NCT03959683

## Introduction

Intracorporeal lithotripters are essential equipment for the success of percutaneous nephrolithotomy (PCNL),^1^ which is the recommended monotherapy for the management of stones >2 cm and lower pole stones >1 cm, that PCNL be the preferred monotherapy because of the highest stone-free rates (SFRs).^1,2^ A variety of lithotripters have been developed that use ballistic, ultrasonic, and laser energy to fragments stones.^3–5^ The ShockPulse-SE (Olympus, Center Valley, PA, USA) lithotripter has been widely adopted since its introduction in the United States in 2017. ShockPulse-SE has a unique ultrasonic generator that produces a ballistic force (300 Hz) and in inanimate studies. Carlos and colleagues demonstrated superior stone clearance compared with the LUS-2™ (Olympus), Cyber-Wand™ (Olympus), and LithoClast™ (Nyon, Switzerland) devices.^6^

In 2018, EMS and Boston Scientific codeveloped and launched a dual-energy (ultrasonic/ballistic 12 Hz) single-probe lithotripter named Trilogy. Multiple bench^6^ and limited surgical evaluations^7–9^ have been published demonstrating exceptional lithotripsy potential; however, a comparison with currently utilized lithotrites has not yet been performed. The purpose of this article is to compare Trilogy with the ShockPulse-SE system in a prospective randomized multicenter clinical trial.

In prior studies,^3,4,10^ the primary metric for lithotripsy efficiency has been stone clearance rate (mm^2^/min). Quantifying stone burden is clinical beneficial for surgical planning and from a research perspective to compare the most efficient modes of stone clearance. In collaboration with the Mayo Clinic–Rochester, MN we incorporate a novel software adjunct to objectively quantify total stone burden (cm^3^) and stone characteristics (HU) on a noncontrasted CT.^11^ This is the first comparative trial, to our knowledge, to use this type of program to measure the stone volume to calculate clearance rates (cm^3^/min) between two lithotripters. We hypothesize that both the Trilogy and ShockPulse-SE lithotripter will demonstrate superior stone clearance rates compared with previous lithotripters and that for larger harder stones, Trilogy will preserve clearance rates compared with the other lithotripters.

## Materials and Methods

After institutional review board approval at all institutions (IRB: 1807352316), we performed a prospective multi-institutional randomized control trial comparing two novel lithotripters: Swiss LithoClast^®^ Trilogy (EMS—Nyon and Boston Scientific Marlborough, MA, USA) and ShockPulse-SE (Olympus). After consent was obtained to undergo PCNL and the surgeon identified a patient as an appropriate candidate for the trial based on stone size and inclusion or exclusion criteria, the patient was enrolled and randomized to a lithotripter by a dedicated clinical research assistant in a 1:1 ratio with either the Trilogy or ShockPulse-SE lithotripter established by our biostatistician using RedCap.

We included patients >18 years with stones ≥20 mm in diameter based on standard preoperative CT imaging or >15 mm in a lower pole stone. Stone size was established by measuring greatest diameter of contiguous stone material in the axial series by the primary surgeon and confirmed to be accurate based on the radiographic read. Stone surface area (SA) (mm^2^) was measured by the surgeon. Preoperative stone characteristics, including stone volume (cm^3^), HU, and number of stones, were objectively recorded using quantitative Stone Analysis Software (qSAS) developed by the CT Clinical Innovation Center (Rochester, MN, USA).^10^

Patient demographics, medical and stone history, perioperative findings, and postoperative outcomes were prospectively recorded in an encrypted RedCap database. Patients were excluded if they were pregnant, had an untreated urinary tract infection, were anticipated to need a multiaccess PCNL, or had prior shockwave lithotripsy within 3 months of anticipated PCNL. Percutaneous renal access was obtained by the urologist unless the patient had an indwelling nephrostomy tube. The decision for location of percutaneous accesses and patient positioning was at the surgeon's discretion.

All procedures were performed by seven surgeons at three high-volume tertiary stone institutions where ShockPulse-SE was the institutional lithotripter of choice for >24 months. Before enrolling in the study, each surgeon was required to have performed at least 10 PCNLs with both lithotripters to ensure proficiency with each device. Permitted access sheaths ranged from 24 to 30F to accommodate the 11.7/9.5-F × 350–440 mm Trilogy, 11.3/9.9-F × 396 mm ShockPulse-SE probes and 24-F rigid nephroscopes. All intraoperative data were entered through an immediate postoperative RedCap survey completed by the operating surgeon. The survey recorded total lithotripsy time, any device malfunctions, and a device assessment, including overall surgeon satisfaction using a 10-point Likert scale.

Stone clearance times were recorded by research personnel with a stopwatch starting from the moment the lithotripter was first activated on the target stone until the stone was removed and the surgeon switched to flexible nephroscopy. In the event lithotripsy was restarted after inspection of the collecting system by flexible nephroscopy; timing resumed. Total operative room (OR) time was calculated using only unilateral percutaneous procedures without pre-existing renal access and was established from the time of intubation to the surgery stop time designated before extubation.

All patients received a postoperative CT scan of the abdomen and pelvis to evaluate for residual stone burden and any injury to surrounding structures. The management of residual stone fragments was left to surgeon preference. Secondary procedures for stone removal were recorded along with postoperative outcomes, including complications using the Clavien–Dindo scale and were classified as immediate postoperative or delayed (90-day) complications. The risk of postoperative infectious complications was assessed using the quick Sequential Organ Failure Assessment (qSOFA) scoring system because of a recent Endourology Disease Group Excellence (EDGE) publication demonstrating a high prediction for infection-related complications after PCNL.^12^

Clinic follow-up appointments at 6–12 weeks provided SFRs through kidney, ureter, and bladder radiograph, and ultrasound and delayed complications. Postoperative stone analyses were designated as hard (Brushite and calcium oxalate monohydrate) or soft (hydroxyapatite, struvite, calcium oxalate dihydrate, and uric acid) based on the >50% stone composition. Statistical analysis was performed by a biostatistician from the department of statistics at Indiana University. Student's *t*-test with a power of 90% and a significance level of alpha = 0.05 was used to perform a power analysis. The primary outcome of interest was the clearance rate of the targeted stone burden. Preliminary *in vitro* data showed a reduction of ∼50% in the mean lithotripter clearance time of a 1 cm^3^ BegoStone for the Trilogy lithotrite compared with the ShockPulse-SE. Given the increased variability of use *in vivo* and across subjects, a more conservative difference was considered for sample size estimation. Assuming a 25% improvement in clearance rate that results in an effect size of 0.7, a sample size of 44 subjects in each group is required. To account for the possibility of unevaluable data a total of 100 patients were enrolled to quantify differences in lithotripsy potential by each lithotripter. A multivariate analysis for the impact stone volume, number, hardness, and type of lithotripter had on clearance time, operative time, and secondary procedures was performed.

## Results

Between February 2019 and June 2020, 100 patients with a target kidney stone ≥1.5 cm underwent PCNL using either Trilogy (*n* = 51) or ShockPulse-SE (*n* = 49). Men represented 39.2% *vs* 32.5% (*p* = 0.678) for each group, respectively. There was no difference in mean age at treatment 59.6 ± 14.8 years *vs* 60.4 ± 16.2 years, and body mass index 33.4 ± 12.3 kg/m^2^ *vs* 32.5 ± 10.1 kg/m^2^ for Trilogy *vs* ShockPulse-SE, respectively (*p* ≥ 0.5). There were no differences with laterality of the procedure between groups. The mean volume of the target stone was 4.18 ± 4.79 cm^3^ *vs* 3.86 ± 3.43 cm^3^ (*p* = 0.713) for Trilogy *vs* ShockPulse-SE. SA (mm^2^) and HU were similar between groups and are reported in Table 1.

**Table 1.** Demographics

	Trilogy (*N* = 51)	ShockPulse (*N* = 49)	p
Age at time of surgery	59.6 ± 14.8	60.4 ± 16.2	0.793
Gender (% male)	39.2% (20)	51.0% (25)	0.315
BMI	33.4 ± 12.3	32.5 ± 10.1	0.678
Has the patient had stone surgery before? (% yes)	41.2% (21)	44.9% (22)	0.840
SWL (%)	15.7% (8)	30.6% (15)	0.098
URS (%)	17.6% (3)	12.2% (6)	0.466
PCNL (%)	5.9% (3)	12.2% (6)	0.313
Right	35.3% (18)	28.6% (14)	
Left	39.2% (20)	36.7% (18)	
Bilateral	25.5% (13)	34.7% (17)	0.594
Study side (% right)	45.1% (23)	46.9% (23)	1.000
Partial or complete staghorn calculi? (%)	43.1% (22)	53.1% (26)	0.423
Total number of discrete stones to treat?	2.39 ± 1.74	2.94 ± 2.09	0.159
Target stone skin to stone distance	14.2 ± 18.1	11.5 ± 3.9	0.315
Max study side stone diameter	23.2 ± 13.1	25.3 ± 10.8	0.404
Max study side long axis	23.5 ± 11.4	22.5 ± 10.1	0.647
Target stone HU	927 ± 386.1	933.1 ± 410.7	0.940
Target stone SA mm^2^	351.3 ± 277.7	375.3 ± 228.8	0.642
Target stone volume (cm^3^)	4.2 ± 4.8	3.9 ± 3.4	0.713
Target stone volume (mm^3^)	4182 ± 4785	3856 ± 3430	0.713

BMI = body mass index; PCNL = percutaneous nephrolithotomy; SA = surface area; SWL = extracorporeal shockwave lithotripsy; URS = ureteroscopy.

Table 2 summarizes perioperative outcomes, including change in hemoglobin (−1.52 ± 1.6 *vs* −1.59 ± 3.26, *p* = 0.407) and transfusion rate (1.96 *vs* 4.08%, *p* = 0.613), for Trilogy and ShockPulse-SE. Stone clearance rates were calculated with both SA (101.3 ± 92.5 mm^2^/min *vs* 83.7 ± 69.3 mm^2^/min, *p* = 0.292) and volume (1.22 ± 1.67 cm^3^/min *vs* 0.77 ± 0.68 cm^3^/min, *p* = 0.054) for Trilogy and ShockPulse-SE, respectively. On a multivariate analysis of clearance time (minutes; Table 4) accounting for stone size, number, hardness, and lithotripter type, Trilogy was found to have a significant reducing effect (−0.450 [−0.110 to −0.788; 95% confidence interval; CI], *p* = 0.021). Similarly, total OR time was shorter (104.4 ± 48.2 minutes *vs* 121.1 ± 59.2 minutes, *p* = 0.126) and with significant effect on multivariate analysis (−36.03 [−6.64 to −65.41; 95% CI], *p* = 0.017) assessing for the same variables as aforementioned.

**Table 2.** Outcomes

	Trilogy (*N* = 51)	ShockPulse (*N* = 49)	p
Outpatient *vs* admitted (% OP)	13.7% (7)	8.2% (4)	0.526
Was this patient on anticoagulation (% yes)	11.8% (6)	18.4% (9)	0.410
Was it discontinued? (% yes)	100%	100%	
Positive preoperative urine culture? (%)	49.0% (25)	55.1% (27)	0.556
Preoperative hemoglobin (g/dL)	12.9 ± 2.0	13 ± 2.1	0.801
Preoperative creatinine (mg/mL)	1.0 ± 0.4	1.0 ± 0.46	0.739
ASA score	2.7 ± 0.5	2.7 ± 0.6	0.802
Was this patient's GU anatomy normal? (% yes)	80.4% (41)	79.6% (40)	1.000
Study side access location (% LP)	38.3% (20)	52.3% (25)	0.210
Duration of case (minutes from induction of anesthesia to end of anesthesia)	104.4 ± 48.2	121.1 ± 59.2	0.126
Was the case aborted? (% yes)	1.9% (1)	2.04% (1)	1.000
Clearance time of targeted stone (minutes)	5.8 ± 6.3	6.7 ± 6.2	0.481
Clearance rate SA (mm^2^/min)	101.3 ± 92.5	83.7 ± 69.3	0.292
Clearance rate volume (cm^3^/min)	1.22 ± 1.67	0.77 ± 0.68	0.054
Average hospital LOS (days)	1.3 ± 1.5	1.3 ± 1.2	0.931
LOS traversed >1 midnight (% no)	31.4% (16)	40.8% (20)	0.302
Residual stone (%)	50.0% (8)	75.0% (15)	
Sepsis (%)	6.3% (1)	5.0% (1)	
Bleeding (%)	—	5.0% (1)	
Respiratory (%)	6.3% (1)	10.0% (2)	
Organ complication (%)	6.3% (1)	5.0% (1)	
Debilitation (%)	12.5% (2)	—	
Placement (%)	18.8% (3)	—	
Postoperative hemoglobin (g/dL)	11.3 ± 1.9	11.4 ± 2.1	0.811
Change in hemoglobin (g/dL)	−1.5 ± 1.6	−1.6 ± 3.3	0.811
Postoperative creatinine (mg/mL)	1.2 ± 0.5	1.2 ± 0.6	0.635
Postoperative WBC	11.2 ± 3.8	12.2 ± 4.2	0.227
Did the patient spike a fever overnight? (% yes)	1.9% (1)	—	
Postoperative qSofa 1	90.0% (45)	77.6% (38)	
Postoperative qSofa 2	10.0% (5)	16.3% (8)	
Postoperative qSofa 3	0	2.1% (1)	
Postoperative qSofa 4	0	0	0.242
Transfusions	2.0% (1)	4.1% (2)	0.614
Postoperative CT stone free? (% yes)	56.0% (28)	42.9% (21)	0.105
<2 mm (%)	18.0% (9)	18.4% (9)	0.878
2–3 mm (%)	8.0% (4)	10.2% (5)	0.779
4–10 mm (%)	12% (6)	22.4% (11)	0.056
>10 mm (%)	6.0% (3)	6.1% (3)	0.824
Postoperative hospital-associated complications	9.8% (5)	10.2% (5)	0.678
I	40.0% (2)	80.0% (4)	
II	40.0% (2)	0% (0)	
IIIa	20.0% (1)	0% (0)	
Iva	0%	20.0% (1)	
Secondary procedure performed for residual stone fragment(s)	17.7% (9)	34.7% (17)	**0.005**
Stone culture positive (%)	45.6% (23)	44.2% (21)	1.000
Stone analysis			0.165
COM	52.0% (26)	32.6% (16)	
COD	—	2.0% (1)	
CaP	32.0% (16)	42.9% (21)	
UA	8.0% (4)	11.1% (5)	
Struvite	6.0% (3)	6.7% (3)	
Brushite	2.0% (1)	6.7% (3)	
Hard or soft stone? (% hard)	52.9% (27)	38.7% (19)	0.167
Outpatient follow-up imaging?			0.676
US and KUB	45.1% (23)	42.9% (21)	
US	27.5% (14)	14.3% (7)	
CT	5.9% (3)	14.3% (7)	
None	21.6% (11)	28.6% (14)	
Did patient come to clinic for follow-up? (% yes)	63.3% (32)	66.7% (33)	0.832
Stone free? (% yes)	90.2% (46)	89.8% (44)	0.758
Size of residual stone?	7.3 ± 4.6	8.5 ± 3.3	0.675
90-Day complications?	11.8% (6)	10.2% (5)	0.850
I	16.7% (1)	20% (1)	
II	33.3% (2)	60% (3)	
IIIa	33.3% (2)	—	
IIIb	16.7% (1)	20% (1)	

Secondary procedures performed was found to be statistically significant with a *p* < 0.05 on *t*-test.

ASA, American Society of Anesthesiology; CaP, calcium phosphate, COD, calcium oxalate dihydrate; COM, calcium oxalate; GU, genitourinary; KUB, kidney, ureter, and bladder radiograph; LOS, length of stay; LP, lower pole; OP, outpatient; UA, uric acid; US, ultrasound; WBC, white blood cells.

**Table 4.** Multivariate Analysis

Effect	Clearance time (minutes)				p
	Estimate	Standard error	Standard error		
Lithotripter (Trilogy)	−0.4489	0.1705	−0.7882	−0.1096	**0.010**
Stone volume	0.09709	0.02079	0.05571	0.1385	**<0.001**
Stone number	0.03103	0.044	−0.05655	0.1186	0.483
Hard stone	0.3006	0.1733	−0.04433	0.6456	0.087
*Effect*	*Operative time (minutes)*				p
Lithotripter (Trilogy)	−36.0259	14.6496	−65.4093	−6.6426	**0.017**
Stone volume	2.4557	1.8295	−1.2138	6.1252	0.185
Stone number	3.0301	3.8704	−4.7331	10.7932	0.437
Hard stone	16.5143	14.9704	−13.5126	46.5411	0.275
*Effect*	*Secondary procedures*				p
	*Odds ratio*		*95% CI*		*0.0053*
Lithotripter (Trilogy)	0.256		0.094	0.694	**0.007**
Stone volume	1.105		0.987	1.237	0.082
Stone number	1.021		0.801	1.301	0.868
Hard stone	1.192		0.445	3.197	0.727
Anticoagulated patient	−0.1289		−0.79	0.54	0.704

Secondary procedures performed was found to be statistically significant with a *p* < 0.05 on *t*-test.

Eleven percent of the cases were completed as an outpatient procedure. Of those admitted, the majority of those discharged were within 24 hours of the primary PCNL (68.6% *vs* 59.2%, *p* = 0.243). Mean length of stay was 1.29 ± 1.47 *vs* 1.27 ± 1.18 (*p* = 0.931) with residual stone fragments on CT >4 mm (16.6% *vs* 24.5%; *p* = 0.302) being the most common reason for prolonged hospitalization in the Trilogy and ShockPulse-SE groups, respectively. Postoperative complications were rare (7.84% [4] *vs* 4.08% [2]; *p* = 0.678 Trilogy and ShockPulse-SE) with one Clavien–Dindo IIIb for a hemothorax requiring an anesthetic for an Interventional Radiology-placed chest tube for 48 hours, and one IVa for respiratory insufficiency requiring reintubation and a 72-hour Intensive Care Unit stay. Complications from the time of discharge to 90 days postop were also rare but did include four (4%) grade IIIa events, including two additional anesthetics to place retrograde stents for a premature dislodged nephrostomy tube and for a persistent urine leak. The remaining two complications included a delayed gastrointestinal bleed and pyelonephritis requiring readmission. Patient follow-up rates (63.3% *vs* 66.7%, *p* = 0.832) and the breakdown of stone types were similar, including stone hardness, and are reported in Table 2. Final SFRs were calculated using postoperative CT, effective stone clearance after secondary stone procedure, and follow-up imaging at 6 weeks, and were estimated at 90.2% and 89.8% for Trilogy *vs* ShockPulse-SE (*p* = 0.758), respectively. Device assessment and surgeon satisfaction are reported in Table 3. There was a higher number of device malfunctions in the ShockPulse-SE group ([17] 34.69%) compared with the Trilogy group [(1) 1.96%], which was statistically significant (*p* ≤ 0.001). There was no difference in overall surgeon satisfaction between devices.

**Table 3.** Device Functionality

	Trilogy (*N* = 51)	ShockPulse (*N* = 49)	p
1 highest ultrasound setting?	97.3 ± 9.7	NA	
1 highest ballistic setting?	7.5 ± 2.6	NA	
1 highest suction setting?	55.3 ± 11.2	NA	
1 highest impact setting?	87.8 ± 20.6	NA	
Did the device malfunction? (% yes)	1.9% (1)	34.7% (17)	**<0.0001**
Hand piece	—	4.1% (2)	0.238
Probe	1.9% (1)	10.2% (5)	0.108
Suction failure	—	30.6% (15)	**<0.0001**
Despite the malfunction, could the case be completed with the device?	100% (51)	94.1% (46)	0.799
What was your overall satisfaction with the lithotripter?	8.7 ± 0.9	8.4 ± 1.8	0.340

Secondary procedures performed was found to be statistically significant with a *p* < 0.05 on *t*-test.

## Discussion

**FIG. 1.**
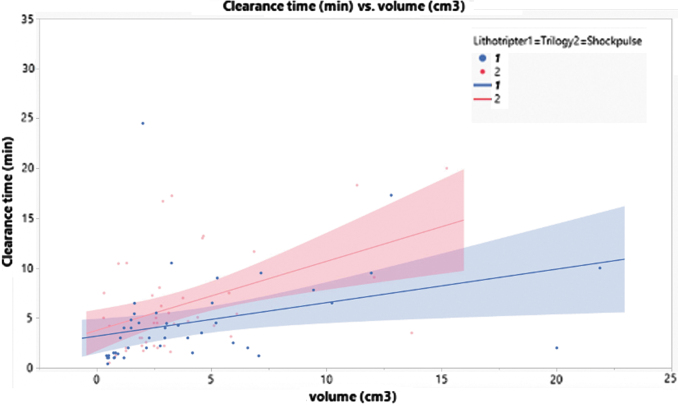
Graphical representation of stone volume (cm^3^) and clearance time (minutes) partitioned by lithotripter type.

The Trilogy and ShockPulse-SE lithotripters represent major technologic advances that improve the efficiency and safe removal of complex calculi from the collecting system. To date there is limited clinical data on both systems^13^ and no head-to-head comparison. This is the first prospective randomized trial to provide clinical data on patient outcomes, surgeon experience, and benefits afforded by either device. The primary endpoint was stone clearance rates. Previous publications on earlier lithotripters suggest a range of stone clearance rates (mm^2^/min), including 16.8–75.9 (LUS-II), 25.9 (StoneBreaker), 31.1–61.9 (Cyberwand), 51.9 (UreTron).^3–5^ Interestingly, ShockPulse-SE has no published data on *in vivo* stone clearance rates, but has been shown in bench models to outperform the aforementioned lithotripters.^14^ In our study, we found clearance rates of 101.3 ± 92.5 mm^2^/min for Trilogy and 83.7 ± 69.3 for ShockPulse-SE (*p* = 0.292); both being far superior to prior studied lithotrites. A recent publication suggested that clearance rates based on stone volume are more representative of stone lithotripsy potential.^8^ Using qSAS to calculate stone volume, we demonstrated clearance rates of 1.22 ± 1.67 and 0.77 ± 0.68 cm^3^/min (*p* = 0.054) for Trilogy *vs* ShockPulse-SE. In addition, we performed a multivariate analysis in which we controlled for stone size and composition, which demonstrated that Trilogy significantly improved clearance time (*p* = 0.021; Table 4). Figure 1 illustrates that, compared with ShockPulse-SE, Trilogy reduces the anticipated increases in clearance times associated with larger stones.

A similar trend of reduced total OR times was seen with Trilogy compared with ShockPulse-SE which was further supported on multivariate analysis (Table 4). Despite all surgeons having used ShockPulse-SE for >2 years and acknowledging that it efficiently clears stones, case completion (104.7 ± 48.2 and 121.1 ± 59.2 minutes; *p* = 0.126) was commonly delayed because of occlusion of the lithotripter's suction system (0% *vs* 30.6%, *p* < 0.001 Trilogy *vs* ShockPulse-SE). Suction occlusion has previously been reported for other lithotripters, including Cyberwand (16%) and LUS-II (3%).^3^ Despite suction malfunctions, 94% of the ShockPulse-SE cases could still be completed with the same lithotripter, which was similar with Trilogy where 100% of cases were completed with the original lithotrite. Aside from delaying case completion, suction occlusion has the potential to increase hematuria or mucosal trauma retropulsed stone fragments leading to residual stone fragments.^8^

A recent publication reported a complication rate of 41.1% after PCNL using the ShockPulse-SE lithotripter, with fever and bleeding accounting for 60.8% and 34.7% of complications, respectively.^10^ Although we observed fewer complications with both lithotripters (Table 2), 40% of the ShockPulse-SE cohort had infectious or bleeding-related complications. To verify these findings, we performed qSOFA assessment of all patients as a predictor of infectious complications after PCNL^12^ and found no difference in scoring between the two cohorts (Table 2). Interestingly, compared with Trilogy, we did see higher rates of secondary procedures for residual stones after ShockPulse-SE lithotripsy (17.7% *vs* 34.7%, *p* = 0.005, respectively). The decision to perform a secondary procedure was at the discretion of the surgeon and could be influenced, but there were more patients with 4–10 mm fragments in the ShockPulse-SE group compared with Trilogy, which is one potential difference accounting for these higher rates. In addition, secondary rates associated with ShockPulse-SE lithotripsy are similar with historical PCNL data (28%–40%),^3–5^ which suggests a potential improvement in fragmentation and extraction with the Trilogy system. One hypothesis is that the suction system integrated into the Trilogy lithotrite minimized fragment propulsion and hematuria, which can obscure vision during PCNL.

Overall surgeon satisfaction with both lithotripters was high (8.69 ± 0.87 and 8.42 ± 1.77, *p* = 0.340; Trilogy *vs* ShockPulse-SE). However, the Trilogy probe has previously been criticized as being heavy leading to decreased surgeon satisfaction.^7^ Concerns over the safety profile of the Trilogy lithotripter were largely mitigated by animal studies by Khoder and colleagues.^15^ Similarly, in our limited medical evaluation, we encountered no tissue damage by the Trilogy lithotripter.^7^ Overall, there were no complications as a direct result of the lithotripter with acceptable SFR of 90.2% and 89.7%, for Trilogy and ShockPulse-SE, respectively.

This study is not without limitations. First, it is multi-institutional trial with multiple surgeons each with different techniques, which could affect our primary outcome measures. We attempted to minimize this variability with our protocol on the specifics for stone removal, definition of stone clearance, and requirement of a postoperative CT. In addition, our device assessment was designed to assess the efficiency of the lithotripter; however, the data from this study do not adequately support subjective ergonomic preferences for each device. Trilogy has a bulky handpiece, but this finding is not supported in the surgeon satisfaction evaluation. The novel software such as qSAS in future comparative stone studies has the potential to standardize sizing variability. We acknowledge the paucity of data on the validity of the software and understand that our conclusions about stone clearance, as a function of stone volume, would become unsupported if qSAS were to be discredited. Finally, there are concerns over any potential bias given the disclosures of all participating surgeons; however, all surgeons are familiar with ShockPulse-SE and sought to provide objective efficiency, and outcomes data for two available lithotripters using novel stone volume analysis software.

In conclusion, both the Trilogy and ShockPulse-SE lithotripters are highly efficient at removing large renal stones. In this study, we noted differences between the two devices, including fewer device malfunctions, when Trilogy device was utilized. The efficiency, safety, and reliability of Trilogy optimizes stone clearance rates and OR times for large renal stones. In addition, software, such as qSAS, should be utilized for future comparative studies on stone clearance rates, as it provides a more accurate measurement of the amount of stone material in the collecting system.

## Abbreviations Used

BMI: body mass index
CT: computed tomography
HU: Hounsfield unit
KUB: kidney, ureter, and bladder radiograph
LOS: length of stay
OR: operating room
PCNL: percutaneous nephrolithotomy
qSAS: quantitative Stone Analysis Software
qSOFA: quick Sequential Organ Failure Assessment
SA: surface area
SFR: stone-free rate

## References

[1] Assimos D, Krambeck A, Miller NL, et al. Surgical management of stones: American Urological Association/Endourological Society Guideline, PART I. J Urol 2016;196:1153–1160. 27238616 10.1016/j.juro.2016.05.090

[2] Assimos D, Krambeck A, Miller NL, et al. Surgical management of stones: American urological association/endourological society guideline, part II. J Urol 2016;196:1161–1169. 27238615 10.1016/j.juro.2016.05.091

[3] Krambeck AE, Miller NL, Humphreys MR, et al. Randomized controlled, multicentre clinical trial comparing a dual-probe ultrasonic lithotrite with a single-probe lithotrite for percutaneous nephrolithotomy. BJU Int 2011;107:824–828. 21355982 10.1111/j.1464-410X.2010.09567.x

[4] York NE, Borofsky MS, Chew BH, et al. Randomized controlled trial comparing three different modalities of lithotrites for intracorporeal lithotripsy in percutaneous nephrolithotomy. J Endourol 2017;31:1145–1151. 28859485 10.1089/end.2017.0436

[5] Borofsky MS, El Tayeb MM, Paonessa JE, et al. Initial experience and comparative efficacy of the UreTron: A new intracorporeal ultrasonic lithotriptor. Urology 2015;85:1279–1283. 26099874 10.1016/j.urology.2015.03.016

[6] Carlos EC, Wollin DA, Brenton B, et al. In vitro comparison of a novel single probe dual-energy lithotripter to current devices. J Endourol 2018;32:534–540. 29649900 10.1089/end.2018.0143

[7] Nottingham CU, Large T, Cobb K, et al. Initial clinical experience with Swiss LithoClast Trilogy during percutaneous nephrolithotomy. J Endourol 2020;34:151–155. 31588790 10.1089/end.2019.0561

[8] Sabnis RB, Balaji SS, Sonawane PL, et al. EMS Lithoclast Trilogy™: An effective single-probe dual-energy lithotripter for mini and standard PCNL. World J Urol 2020;38:1043–1050. 31177306 10.1007/s00345-019-02843-2

[9] Panthier F, Doizi S, Illoul L, et al. Developing free three-dimensional software for surgical planning for kidney stones: Volume is better than diameter. Eur Urol Focus 2020;S2405-4569:30161–30169. 10.1016/j.euf.2020.06.003 32591284

[10] Yadav BK, Basnet RB, Shrestha A, et al. Comparison between shockpulse and pneumatic lithotripsy in percutaneous nephrolithotomy. World J Urol 2020;39:1–5. 10.1007/s00345-020-03239-3 32448972

[11] Duan X, Wang J, Qu M, Leng S, Liu Y, Krambeck A, McCollough C. Kidney stone volume estimation from computerized tomography images using a model based method of correcting for the point spread function. J Urol 2012;188:989–995. 22819107 10.1016/j.juro.2012.04.098 PMC3927405

[12] Yaghoubian A, Batter T, Mozafarpour S, et al. Use of the quick sequential organ failure assessment score for prediction of intensive care unit admission due to septic shock after percutaneous nephrolithotomy: A multicenter study. J Urol 2019;202:314–318. 30829131 10.1097/JU.0000000000000195

[13] Axelsson TA, Cracco C, Desai M, et al. Consultation on kidney stones, Copenhagen 2019: Lithotripsy in percutaneous nephrolithotomy. World J Urol 2020;7:1–8. 10.1007/s00345-020-03383-w PMC8217030 32728884

[14] Chew BH, Matteliano AA, de Los Reyes T, et al. Benchtop and initial clinical evaluation of the ShockPulse Stone Eliminator in percutaneous nephrolithotomy. J Endourol 2017;31:191–197. 27863458 10.1089/end.2016.0664

[15] Khoder W, Strittmatter F, Alghamdi A, et al. Comparative evaluation of tissue damage induced by ultrasound and impact dual-mode endoscopic lithotripsy versus conventional single-mode ultrasound lithotripsy. World J Urol 2019;38:1051–1058. 31144092 10.1007/s00345-019-02747-1

